# bNEAT: a Bayesian network method for detecting epistatic interactions in genome-wide association studies

**DOI:** 10.1186/1471-2164-12-S2-S9

**Published:** 2011-07-27

**Authors:** Bing Han, Xue-wen Chen

**Affiliations:** 1 Bioinformatics and Computational Life Sciences Laboratory, ITTC, Department of Electrical Engineering and Computer Science, The University of Kansas, 1520 West 15th Street, Lawrence, KS 66045, USA

## Abstract

**Background:**

Detecting epistatic interactions plays a significant role in improving pathogenesis, prevention, diagnosis and treatment of complex human diseases. A recent study in automatic detection of epistatic interactions shows that Markov Blanket-based methods are capable of finding genetic variants strongly associated with common diseases and reducing false positives when the number of instances is large. Unfortunately, a typical dataset from genome-wide association studies consists of very limited number of examples, where current methods including Markov Blanket-based method may perform poorly.

**Results:**

To address small sample problems, we propose a Bayesian network-based approach (bNEAT) to detect epistatic interactions. The proposed method also employs a Branch-and-Bound technique for learning. We apply the proposed method to simulated datasets based on four disease models and a real dataset. Experimental results show that our method outperforms Markov Blanket-based methods and other commonly-used methods, especially when the number of samples is small.

**Conclusions:**

Our results show bNEAT can obtain a strong power regardless of the number of samples and is especially suitable for detecting epistatic interactions with slight or no marginal effects. The merits of the proposed approach lie in two aspects: a suitable score for Bayesian network structure learning that can reflect higher-order epistatic interactions and a heuristic Bayesian network structure learning method.

## Background

Genome-wide association study (GWAS) focuses on studies of the genetic variants related with a variety of diseases from individual to individual among a cohort of cases (people with the disease) and controls (similar people without the disease) [1-3]. The most important category of genetic variations is SNP (Single Nucleotide Polymorphism), which influences disease risk. Conventional analysis methods for GWAS data only consider one SNP at a time by the Armitage trend test (ATT) and are likely to miss genetic variants having slight to moderate marginal effects but strong joint effects on disease risk. Moreover, it is widely acknowledged that some common complex diseases such as various types of cancers, cardiovascular disease, and diabetes are caused by multiple genetic variants [4]. Therefore, there is an urgent need to detect high-order epistasis (gene-gene interaction), which refers to the interactive effect of two or more genetic variants on complex human diseases, and explore how these epistatic interactions confer susceptibility to complex diseases [5]. However, the very large number of SNPs checked in a typical GWAS (more than 10 million) and the enormous number of possible SNP combinations make detecting high-order epistatic interactions from GWAS data statistically and computationally challenging [6,7].

During the past decade, some heuristic computational methods have been proposed to detect causal interacting genes or SNPs. One type of computational methods for epistatic interactions detection are statistical methods including multifactor dimensionality reduction (MDR) [8-11], penalized logistic regression (stepPLR [12], lassoPLR [13]), and Bayesian epistasis association mapping (BEAM) methods [14]. MDR is a non-parametric and model-free method based on constructing a risk table for every SNP combination [11]. If the case and control ratio in a cell of this risk table is larger than 1, MDR will label it as “high risk”, otherwise, “low risk”. By the risk table, MDR can predict disease risk and will select the SNP combination with the highest prediction accuracy. StepPLR and lassoPLR make some modifications to avoid the overfitting problem of standard logistic regression when detecting epistatic interactions [15]. For example, stepPLR combines the LR criterion with a penalization of the L2-norm of the coefficients. This modification makes stepPLR more robust to high-order epistatic interactions [12]. In general, most statistical methods can only be applied to small-scale analysis (i.e., a small set of SNPs) due to their computational complexity. Moreover, MDR, stepPLR and lassoPLR are all predictor-based methods, which make them easy to include false positives. Comparing to MDR, stepPLR and lassoPLR, BEAM is a scalable and non-predictor-based statistical method [14]. BEAM partitions SNPs into three groups: group 0 is for normal SNPs, group 1 contains disease SNPs affecting disease risk independently, and group 2 contains disease SNPs that jointly contribute to the disease risk (interactions). Give a fixed partition, BEAM can get the posterior probability of this partition from SNP data based on Bayes theory. A Markov Chain Monte Carlo method is used to reach the optimal SNP partition with maximum posterior probability in BEAM. One drawback of BEAM is that identifying both single disease SNP and SNP combinations simultaneously make BEAM over-complex and weakens its power.

An alternative approach is machine learning based methods, which are based on binary classification (prediction) and treat cases as positives and controls as negatives in SNP data. Support vector machine-based approaches [16] and random forest-based approaches [17] are two commonly-used machine learning methods for epistatic interactions detection. They use SVM or random forest as a predictor and select a set of SNPs with the highest prediction/classification accuracy by feature selection. Like predictor-based statistical methods, machine learning-based methods lack the capability of detecting causal elements and tend to introduce many false positives, which may result in a huge cost for further biological validation experiments [18].

Recently, we propose a new Markov Blanket-based method, DASSO-MB, to detect epistatic interactions in case-control studies [18]. The Markov Blanket is a minimal set of variables, which can completely shield the target variable from all other variables based on Markov condition property. Thus, DASSO-MB can detect the SNP set that shows a strong association with diseases with the fewest false positives. Furthermore, the heuristic search strategy in DASSO-MB can avoid the time-consuming training process as in SVMs and Random Forests.

In this paper, we address the problems by introducing a Bayesian networks-based method, which also employs a Branch-and-Bound technique to detect epistatic interactions. Bayesian networks provide a succinct representation of the joint probability distribution and conditional independence among a set of variables. In general, a structure learning methods for Bayesian networks first defines a score reflecting the fitness between each possible structure and the observed data, and then searches for a structure with the maximum score. Comparing to Markov Blanket based methods, the merits of applying Bayesian networks method to epistatic interaction detection includes: (1) BDE, BIC or MDL scores for Bayesian network structure learning can reflect higher-order interactions and are not sample-consuming; and (2) heuristic Bayesian network structure learning method can solve the classical XOR problem, which may hinder the applications of Markov blanket based approaches.

We apply the bNEAT (Bayesian Networks based Epistatic Association sTudies) method to simulated datasets based on four disease models and a real dataset (the Age-related Macular Degeneration (AMD) dataset). We demonstrate that the proposed method outperforms Markov Blanket methods and other commonly-used methods, especially when the number of samples is small.

## Results

### Analysis of simulation data

We first evaluate the proposed bNEAT method on simulated data sets, which are generated from three commonly used two-locus epistatic models in [15] and one three-locus epistatic model developed in [14]. Model-1 is a multiplicative model, model-2 demonstrates two-locus interaction multiplicative effects and model-3 specifies two-locus interaction threshold effects. There are three disease loci in model-4 [14]. Some certain genotype combinations can increase disease risk in model-4 and there are almost no marginal effects for each disease locus.

To compare the performance of different methods, we use the same data generation process and the similar parameter settings as in [14,15,18]. We generate 50 datasets and each contains 100 markers genotyped for 1,000 cases and 1,000 controls. To measure the performance of each method, we use “power” as the criterion function. Power is calculated as follows:(1)

where *N* is the total number of simulated datasets and *N_D_* is the number of simulated datasets in which all disease associated markers are identified without any false positives.

We compare the bNEAT algorithm with four methods: BEAM, Support Vector Machine, MDR and DASSO-MB on the four simulated disease models. The BEAM software is downloaded from http://www.fas.harvard.edu/~junliu/BEAM and we set the threshold of the B statistic as 0.1 [14]. For support vector machines, we use LIBSVM with a RBF kernel to detect gene-gene interactions and the detail is shown in [18]. Since MDR algorithm can not be applied to a large dataset directly, we first reduce the number of SNPs to 10 by ReliefF [19], a commonly-used feature selection algorithm, and then MDR performs an exhaustive search for a SNP set that can maximize cross-validation consistency and prediction accuracy. For DASSO-MB, we set the threshold of *G*^2^ test as 0.01 to determine (conditional) dependence and (conditional) independence.

**Figure 1:**
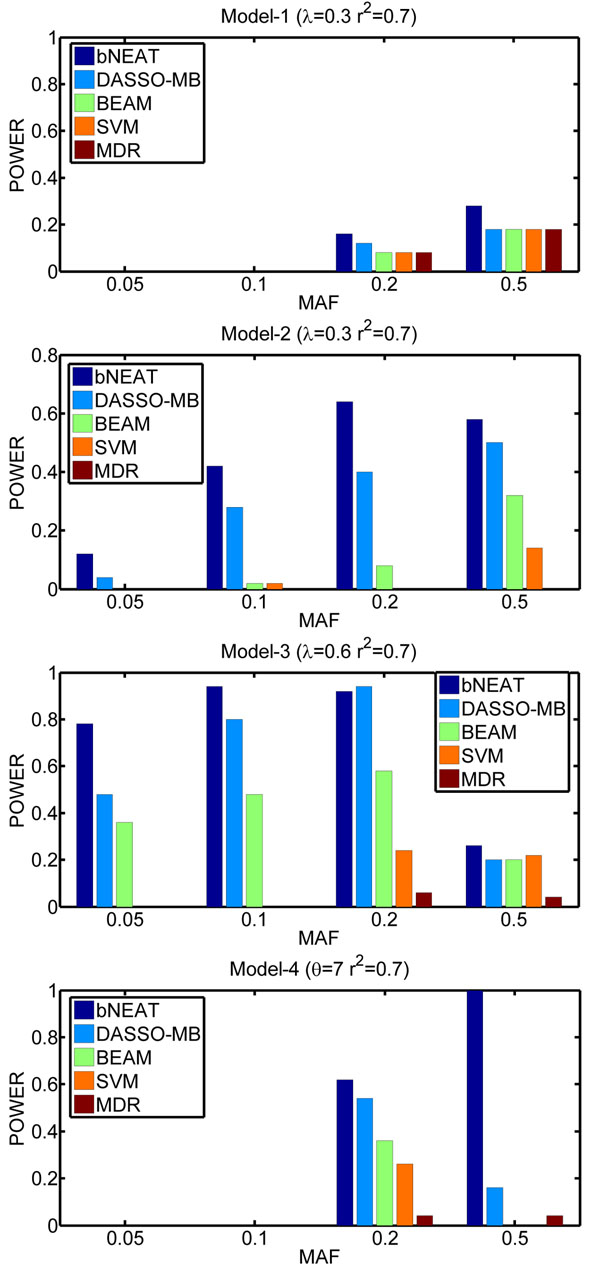
**Performance comparison for *r*^2^ = 0.7** The power is defined as the proportion of simulated datasets whose result only contains disease associated markers without any false positives.

**Figure 2:**
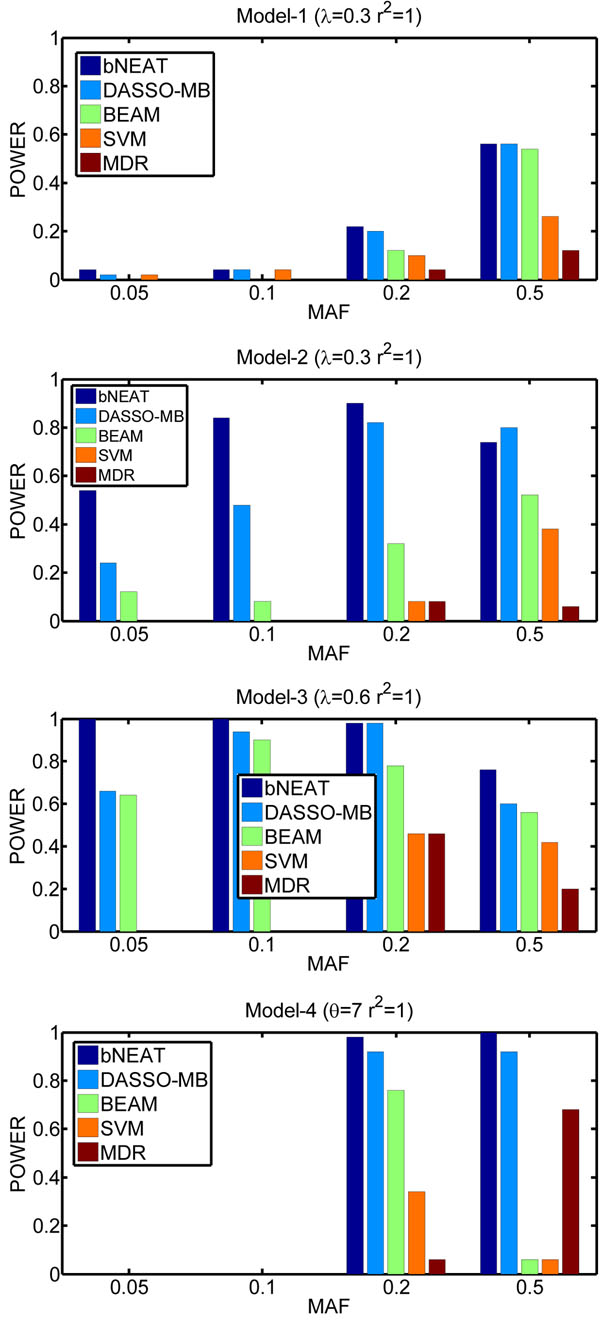
**Performance comparison for *r*^2^ = 1** The power is defined as the proportion of simulated datasets whose result only contains disease associated markers without any false positives.

The results on the simulated data are shown in Figures 1 and 2. As can be seen, among the five methods, the bNEAT algorithm performs the best. BEAM is worse than both bNEAT and DASSO-MB. One possible reason is that BEAM tries to detect single disease loci and epistatic interactions simultaneously. This strategy is unnecessary and makes BEAM over-complex. The other possible reason is that BEAM uses fixed Dirichlet priors in its Bayesian marker partition model, which may not reflect and penalize the model complexity appropriately [20].

Typically, GWAS can not generate a large number of samples due to the high experiment cost. Thus, the performance of various computational methods for epistatic interaction detection in case of small samples is important. We explore the effect of the number of samples on the performance of bNEAT, DASSO-MB, BEAM and SVM. We generate synthetic datasets containing 40 markers genotyped for different number of cases and controls with *r*^2^ = 1 and MAF=0.5.

**Figure 3:**
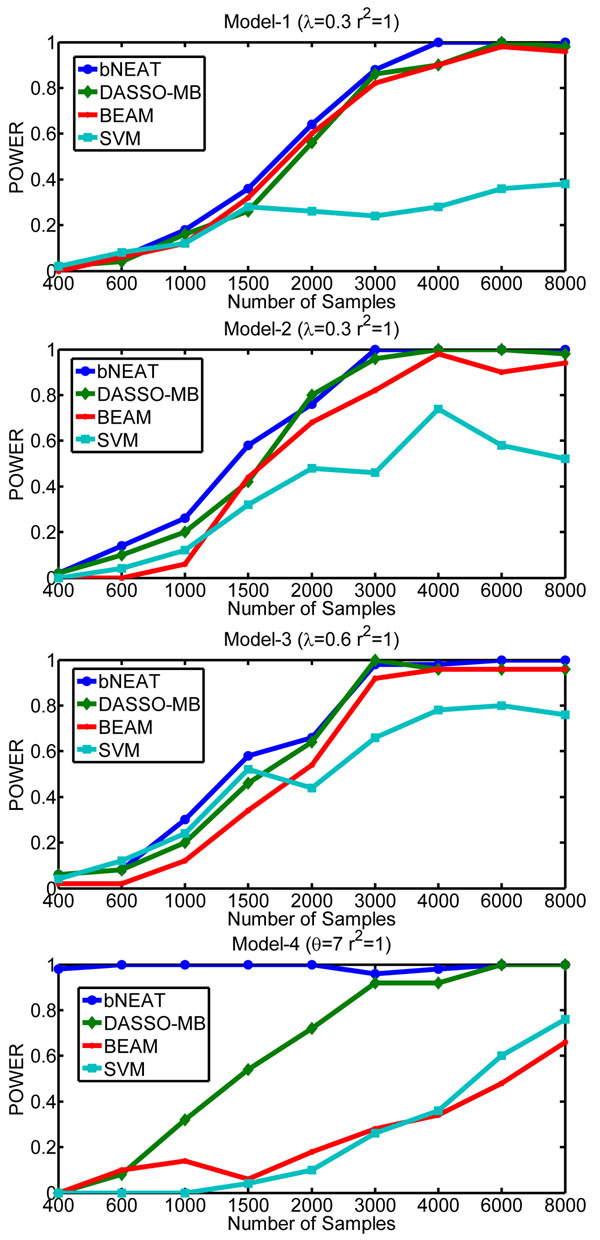
Comparison of sample efficiency

The results are shown in Figure 3. We find that bNEAT is more sample-efficient than other methods in that it can achieve the highest power when the number of samples is the same. In addition, it needs fewer samples to reach the perfect power comparing to other methods. DASSO-MB is the second best. For models 1-3, almost all methods can obtain a perfect power except SVM when the number of samples is larger than 4000. SVM can not achieve a perfect power even though we have sufficient samples (≥ 8000). This may indicate that the predictor-based methods lack the ability to find causal elements precisely. The result from model-4 is particularly interesting: bNEAT exhibits overwhelming superiority over other three methods, as bNEAT yields a perfect power even the number of samples is small (around 400), which indicates that bNEAT is especially suitable for detecting epistatic interactions with slight or no marginal effects.

### Results on AMD data

In this section, we apply bNEAT to large-scale (large number of SNPs but small samples) datasets in real genome-wide case-control studies, which often require genotyping of 30,000–1,000,000 common SNPs. We make use of an Age-related Macular Degeneration (AMD) dataset containing 116,204 SNPs genotyped with 96 cases and 50 controls [21]. Multiple genetic factors cause AMD, which can result in a loss of vision.

To remove inconsistently genotyped SNPs, we perform filtering process as in [18]. After filtering, there are 97,327 SNPs remained. Since the number of SNPs is very large, restricting the search space to avoid unreasonable search by selecting some candidate SNPs as in [22] is necessary. We select top 200 candidate SNPs based on *G*^2^ test and then use bNEAT to identify disease SNPs related with AMD. bNEAT detects three associated SNPs: rs380390, rs3913094 and rs10518433. The first SNP, rs380390, is already found in [21] with a significant association with AMD. Although no evidences were reported with the other two SNPs related to AMD in the literature, they may be plausible candidate SNPs associated with AMD.

## Conclusions and discussion

Comparing with many computational methods used for identification of epistatic interactions, Markov Blanket based method can increase power and reduce false positives. However, Markov Blanket based method is sample-consuming and the greedy searching strategy in Markov Blanket method is not suitable for detecting some interaction models with no independent main effects for each disease locus. In this paper, we propose a Bayesian networks method based on Branch-and-Bound technique (bNEAT) to detect epistatic interactions. We demonstrate that the proposed bNEAT method significantly outperforms Markov Blanket method and other commonly-used methods, especially when the number of samples is small.

Even though the bNEAT method is more powerful than Markov Blanket based method, it can not be directly applied to genome-wide dataset due to the large number of SNPs. Integrating Markov chain Monte Carlo or simulated annealing technique into our bNEAT method to make it scalable to genome-wide dataset is one direction for future research. Moreover, we will explore different score schemes for epistatic interaction detection by Bayesian networks. For example, information-based score schemes (e.g., AIC score and BIC score) are derived in case of large number of samples [23]. When the number of samples is small, the approximation in the inference of both AIC score and BIC score can not hold any more. In fact, the penalty term for model complexity in AIC score and BIC score can also reflect the variance of the model [24]. Thus in our future work, we will design a new score scheme by estimating the penalty term from data to make sure that the score scheme can fit data better.

## Methods

### Bayesian networks

A Bayesian network is a directed acyclic graph (DAG) *G* consisting of nodes corresponding to a random variable set *X* = {*X*_1_, *X*_2_, …, *X_n_*} and edges between nodes, which determine the structure of *G* and therefore the joint probability distribution of the whole network [25].

**Definition 1 (Conditional Independence)***For three random variables (nodes) X*, *Y and Z*, *if the probability distribution of X conditioned on both Y and Z is equal to the probability distribution of X conditioned only on Y*, *i.e.*, *P*(*X *|* Y*, *Z*) = *P*(*X *|* Y*), *X is conditionally independent of Z given Y.*

This conditional independence is represented as . Similarly,  represents conditional dependence [26].

**Theorem 1 (Local Markov Assumption)***Each variable is conditionally independent of its nondescendants*, *given its parents in the DAG G.*

By applying the local Markov assumption, the joint probability distribution *J* can be represented as(2)

where *Pa*(*X_i_*) denotes the set of parents of *X_i_* in *G* . Therefore, there are two components in a Bayesian network. The first component is the DAG *G* reflecting the structure of the network. The second component, *θ*, describes the conditional probability distribution *P*(*X_i_* | *Pa*(*X_i_*)) to specify the unique distribution *J* on *G*.

**Definition 2 (V-structure)***For three nodes X*, *Y and Z in a Bayesian network*, *a structure with the form of X→Z←Y (no edge between X and Y) is called a v-structure.*

**Definition 3 (D-seperation)***For three nodes X*, *Y and Z in a Bayesian network*, *if there is no active path between X and Y given Z*, *we say that X and Y are d-seperated given Z*, *denoted as Dsep(X*;*Y* | *Z).*

**Figure 4:**
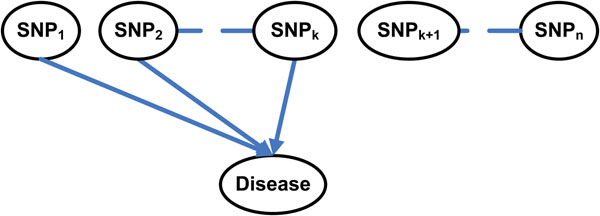
**An Example of Genome-wide Association Studies.** The goal of genome-wide association studies is to identify the *k*-way interaction among SNPs: SNP_1_, SNP_2_,…,SNP_k_, which are associated with disease.

Bayesian networks allow us to explore causal relationships to perform explanatory analysis and make predictions. As shown in Figure 4, GWAS attempts to identify the *k*-way interaction among SNPs: SNP_1_, SNP_2_,…, SNP_k_, which are associated with a disease. The *n* SNP nodes and the disease status/label node construct a Bayesian network and we want to determine which SNP nodes are the parent nodes of the disease status /label node.

### Structure learning of Bayesian networks

Even though a Bayesian network can be constructed by an expert, most tasks of determining the network structure are too complex for humans. We have no choice but to learn the network structure and parameters from data. There are two types of structure learning methods for Bayesian networks: constraint-based methods and score-and-search methods.

The constraint-based methods first build the skeleton of the network (undirected graph) by a set of dependence and independence relationships. Next constraint-based methods direct links in the undirected graph to construct a directed graph with d-separation properties corresponding to the dependence and independence determined [27-29]. Even though constraint-based methods are developed with a rigorous theoretical foundation, errors in conditional dependence and independence will affect the stability of constraint-based methods and this problem is especially serious when the number of samples is small.

The score-and-search methods view a Bayesian network as a statistical model and transform the structure learning of Bayesian network into a model selection problem [30]. To select the best model, a score function is needed to indicate the fitness between a network and the data. Then the learning task is to find the network with the highest score. Thus, score-and-search methods typically consist of two components, (1) a score function, and (2) a search procedure. In this paper, we focus on structure learning approaches for Bayesian networks based on score-and-search methods because score-and-search methods are more robust for small data sets than constraint-based methods.

One of the most important issues in score-and-search methods is the selection of score function. A natural choice of score function is the likelihood function. However, the maximum likelihood score often overfits the data because it does not reflect the model complexity. Therefore, a good score function for Bayesian networks’ structure learning must have the capability of balancing between the fitness and the complexity of a selected structure. There are several existing score functions based on a variety of principles, such as the information theory and minimum description length (BIC score, AIC score, MDL score) [31-33] and Bayesian approach (BDe score) [34].

The general idea of BDe score is to compute the posterior probability distribution. Consider that we want to learn the structure *S* of a Bayesian network containing *n* nodes from a dataset *D* with *N* examples, and let *q_i_* denote the number of configurations of the parent set *Pa*(*X_i_*) of *X_i_* and let *r_i_* represent the number of states of *X_i_*, the BDe score is obtained as(3)

where *N_ijk_* is the number of cases for *X_i_* in its *k*th configuration and *Pa*(*X_i_*) in the *j*th configuration and . *a_ij_* and *a_ijk_* are user-determined Dirichlet priors which reflect a user’s prior knowledge and we often set *a_ijk_* = *N*/(*r_i_q_i_*). BDe score can penalize the structure complexity inherently by integrating  and measuring the average expected likelihood over different possible choices of  ( is an estimate of parameters from the maximum likelihood method for the structure *S*) [35,36]. For AIC (*Akaike information criterion*) score and BIC (*Bayesian information criterion*) score, we can write a general score scheme as:(4)(5)

where *C*(*S*) represents the structure complexity [32] . The first term of this score scheme measures the fitness between the structure, and data and the second term reflects structure complexity. With a maximum likelihood method, we can get(6)

In (4), by setting *f*(*N*) = 1, we get the AIC score as(7)

If we set *f*(*N*) = 1/2log(N), we get the BIC score, which is(8)

The BIC score are derived from a Taylor expansion and Laplace approximation when the number of samples *N* approaches ∞. This results in a problem that the structure penalty term in (8) is very strict when the number of samples is small; therefore, we adjust the coefficient of the second term in (8) from 1/2 to a smaller number (in our applications, we empirically set it to be 0.17 for all the datasets we study).

The computational task in score-and-search methods is to find a network structure with the highest score. The searching space consists of a superexponential number of structures-2*^O^*^(^^*n*^2^)^ and thus exhaustively searching optimal structure from data for Bayesian networks is NP-hard [37]. One simple heuristic search algorithm is greedy hill-climbing algorithm. In greedy hill-climbing algorithm, there are three types of operators that change one edge at each step:

• Add an edge

• Remove an edge

• Reverse an edge

By these three operators, we can construct the local neighbourhood of current network. Then we select the network with the highest score in the local neighbourhood to get the maximal gain. This process can be repeated until it reaches a local maximum. However, greedy hill-climbing algorithm cannot guarantee a global maximum [30]. Other structure learning methods for Bayesian networks include Branch-and-Bound (B&B) [38,39], genetic algorithms [40] and Markov chain Monte Carlo [41]. Branch-and-Bound algorithms guarantee the optimal results in a significantly reduced search time compared to exhaustive search. Thus, we will employ B&B algorithms in our study.

**Figure 5:**
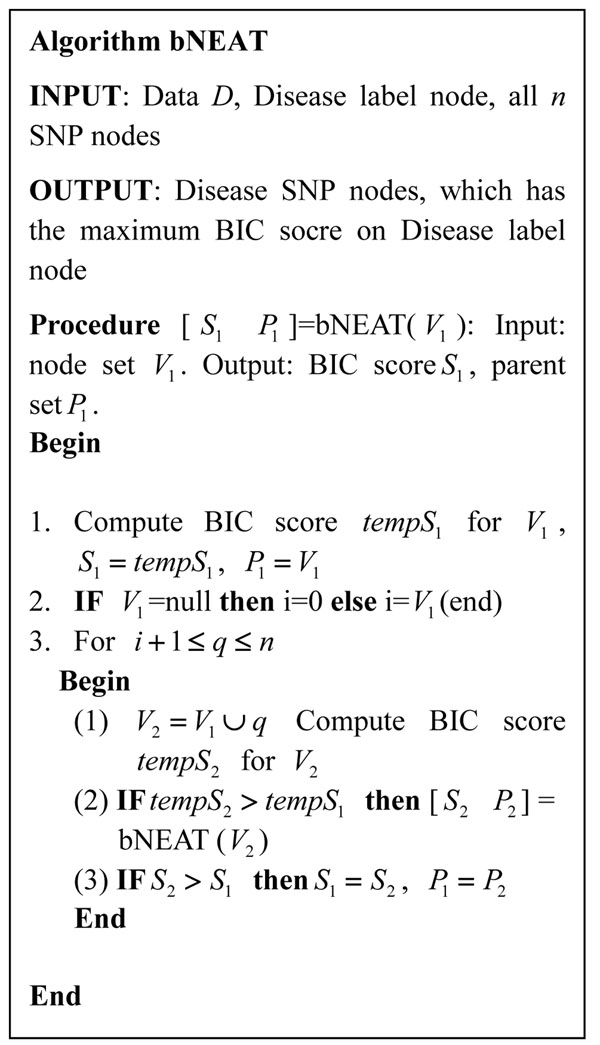
the bNEAT algorithm

The proposed method uses B&B to search a structure that maximizes the BIC score. The algorithm is shown in Figure 5. bNEAT starts from an empty node set and constructs a depth-first search tree to find the optimal parent (disease SNPs) set for the disease label node. In our B&B search, instead of using the pruning strategy as in [38,39], which sets a lower bound for the MDL score to prune the search tree, we stop the recursive calls when we observe that the BIC score will decrease on the children state of the current state. This strategy cannot guarantee global optima theoretically. However, it will significantly speed up the search process.

## Competing interests

Authors declare that they have no competing interests.

## Authors' contributions

BH designed and implemented the algorithm. XWC conceived the study and designed the experiments. Both authors drafted the manuscript and approved the final manuscript.
